# Exoskeletons and economics: indoor arthropod diversity increases in affluent neighbourhoods

**DOI:** 10.1098/rsbl.2016.0322

**Published:** 2016-08

**Authors:** Misha Leong, Matthew A. Bertone, Keith M. Bayless, Robert R. Dunn, Michelle D. Trautwein

**Affiliations:** 1 California Academy of Sciences, San Francisco, CA, USA; 2 Department of Entomology and Plant Pathology, North Carolina State University, Raleigh, NC, USA; 3 Department of Applied Ecology, North Carolina State University, Raleigh, NC, USA; 4 Center for Macroecology, Evolution and Climate, Natural History Museum of Denmark, University of Copenhagen, Copenhagen, Denmark

**Keywords:** biodiversity, socioeconomics, income, urban ecosystem, indoor biome, landscape ecology

## Abstract

In urban ecosystems, socioeconomics contribute to patterns of biodiversity. The ‘luxury effect’, in which wealthier neighbourhoods are more biologically diverse, has been observed for plants, birds, bats and lizards. Here, we used data from a survey of indoor arthropod diversity (defined throughout as family-level richness) from 50 urban houses and found that house size, surrounding vegetation, as well as mean neighbourhood income best predict the number of kinds of arthropods found indoors. Our finding, that homes in wealthier neighbourhoods host higher indoor arthropod diversity (consisting of primarily non-pest species), shows that the luxury effect can extend to the indoor environment. The effect of mean neighbourhood income on indoor arthropod diversity was particularly strong for individual houses that lacked high surrounding vegetation ground cover, suggesting that neighbourhood dynamics can compensate for local choices of homeowners. Our work suggests that the management of neighbourhoods and cities can have effects on biodiversity that can extend from trees and birds all the way to the arthropod life in bedrooms and basements.

## 1. Introduction

In cities, humans exert a strong effect on biodiversity. In addition to being influenced by gradients in climate or habitat, biodiversity in cities can also be strongly influenced by socioeconomics. Affluence, along with its many associated phenomena, tends to have a positive effect on biodiversity, a so-called luxury effect [1–4]. Patterns of greater species richness in higher-income neighbourhoods have been demonstrated for both plants and animals, including birds [5], lizards [6] and bats [7]. The first studies of the luxury effect focused on plants and found an increase in plant diversity at the scale of neighbourhoods associated with higher income [1,2]. Plants in urban areas, more so than animals, have a direct link to socioeconomics as vegetative landscaping is dependent on human decision-making and financial resources. Plant coverage and diversity can then directly influence animal diversity (including that of arthropods) through the provision of food resources and habitats [8]. The diversity of birds, for example, has been shown to be associated with affluence, with high vegetation cover as an explanatory mechanism [5].

In contrast to the recognized pattern of increased biodiversity in wealthier neighbourhoods, there is a general perception that homes in poorer neighbourhoods harbour more indoor arthropods [9,10]. The ecology of the indoor biome is relatively unexplored, yet recent work has revealed that it harbours more biodiversity than previously recognized [11]. Our own research characterizing indoor arthropods revealed that the average home contains more than a hundred arthropod species [12]; the vast majority of these species being non-pests.

Here, we expand our focus on arthropods in the indoor environment, beyond the pest groups traditionally studied by urban entomologists, to examine indoor biodiversity patterns from a local and landscape perspective. Using data from our previous survey of arthropods inside 50 houses [12], we ask how the surrounding landscape context, both ecologically and economically, influences the diversity and composition of arthropods inside homes. By building upon previous studies of the luxury effect, we explore whether socioeconomic factors that have been found to drive plant coverage and diversity outdoors influence the prevalence of arthropods that find their way indoors.

## 2. Methods

Our study system was located in and around Raleigh, North Carolina in the southeastern United States. We thoroughly sampled all living and dead arthropods found inside 50 homes within a 65 km radius of central Raleigh through active searching and hand collecting (further details in reference [12]). We collected the specimens to represent all morphotypes in a house, but not their abundance (e.g. we did not collect all ants observed if they were the same species). Specimens were identified to the family level, and we therefore use the term ‘arthropod diversity’ throughout to refer to number of arthropod families. No spatial autocorrelation was detected for house arthropod diversity or each landscape variable when assessed by Mantel tests in R package ‘*ade4*’ [13]. All analyses were performed in R 3.2.0 [14].

We considered several biological, geophysical and socioeconomic variables at local and landscape scales that we hypothesized could influence indoor arthropod diversity (table 1), and scaled them from 0 to 1. We then created a correlation matrix with these initial 12 variables with R package *corrplot* [15] (electronic supplementary material, figure S1). We found some variables to be highly correlated with one another (Pearson's *r* > |0.5|), so restricted our analyses to seven factors that maximized coverage and questions of interest. On the landscape scale, we included remotely sensed canopy cover within a 100 m radius, remotely sensed impervious surface area within a 500 m radius, and mean neighbourhood income at the census block level. On the scale of each property's extent, we included house age, local ground vegetation diversity, local ground vegetation cover and local canopy cover. We suspected house square footage of being linked with sampling effort, so included it in the model to account for potential bias. We then used R package *glmulti*, which does automated model selection with generalized linear models (GLMs) [16], to generate GLMs of all possible model combinations with a Poisson distribution (with house arthropod diversity as the response variable and the explanatory variables listed above) and automatically select the best model based on Akaike information criterion (AIC) and Bayes information criterion (BIC) scores. Then, with the most critical environmental variables identified, we tested for the effect of interactions among those that made biological sense.

**Table 1.** Biological, geophysical and socioeconomic variables. All of these variables were considered for inclusion in the analyses. Based on a correlation matrix with these initial 12 variables (electronic supplementary material, figure S1), we found some to be highly correlated with one another (Pearson's *r* > |0.5|), so we restricted our analyses to those factors that maximized coverage and questions of interest. These variables are indicated in the ‘used’ column below. sp., species.

code	variable	scale	used	details
groundDiv	local ground vegetation diversity	local (house property extent)	yes	assessed as low (0–5 sp.), medium (6–15 sp.) or high (>15 sp.); for plants <1.5 m tall
canopyDiv	local canopy diversity	local (house property extent)	no	assessed as low (0–5 sp.), medium (6–15 sp.) or high (>15 sp.); for plants >1.5 m tall
groundCover	local ground vegetation cover	local (house property extent)	yes	assessed as low (0–33%), medium (34–66%) or high (67–100%)
canopyCover	local canopy cover	local (house property extent)	yes	assessed as low (0–33%), medium (34–66%) or high (67–100%)
houseAge	house age	local (house property extent)	yes	public property records obtained through online realty website (trulia.com), as of 2015
totalValue	house value	local (house property extent)	no	public property records obtained through online realty website (trulia.com), as of 2015
sqFeet	house square footage	local (house property extent)	yes	public property records obtained through online realty website (trulia.com), as of 2015
income	mean neighbourhood household income	landscape (census block)	yes	American Community Survey 2011 dataset at the census block level, obtained through R package ‘acs’
imp100m	impervious surface area	landscape (100 m radius)	no	National Land Cover Database 2011, accessed through the Multi-Resolution Land Characteristics Consortium (MRLC)
imp500m	impervious surface area	landscape (500 m radius)	yes	National Land Cover Database 2011, accessed through the Multi-Resolution Land Characteristics Consortium (MRLC)
can100m	canopy cover	landscape (100 m radius)	yes	National Land Cover Database 2011, accessed through the Multi-Resolution Land Characteristics Consortium (MRLC)
can500m	canopy cover	landscape (500 m radius)	no	National Land Cover Database 2011, accessed through the Multi-Resolution Land Characteristics Consortium (MRLC)

We also explored how different arthropod taxonomic groups may be differentially affected by these variables. We tested for variation of the indoor arthropod community composition with the above-mentioned variables by conducting a permutational multivariate analysis of variance [17] test based on Bray–Curtis dissimilarity with 9999 permutations. These analyses were performed using the *adonis* function in R package *vegan* [18].

Our dataset used in these analyses has been uploaded as part of the electronic supplementary material.

## 3. Results and discussion

**Figure 1.**
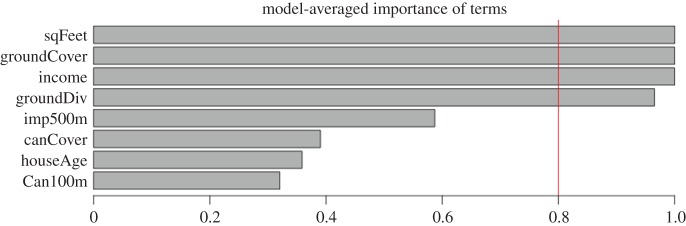
Model-averaged importance of terms (calculated by the sum of the Akaike weights for all models). House square footage (sqFeet), local ground vegetation cover (groundCover), mean neighbourhood income (income) and local ground vegetation diversity (groundDiv) were the most important variables for predicting indoor arthropod diversity. (Online version in colour.)

Here, we found that indoor arthropod diversity was best predicted by models that take into consideration not only house square footage, local ground vegetation cover and diversity, but also mean neighbourhood income (table 2*a,b* and figure 1). Although we expected that indoor arthropod diversity would increase both with house size and with surrounding plant coverage and diversity in local gardens, we were surprised by the strong support for a persistent and significant positive association of arthropod diversity with mean neighbourhood income.

**Table 2.** Best model summary output tables. Pseudo-*R*^2^ calculated as 1 – (residual deviance/null deviance): (*a*) based on AIC score; (*b*) based on BIC score and (*c*) with interaction term.

variable	estimate	s.e.	*z*-value	*p*-value
(*a*) AIC = 548.9029; pseudo-*R*^2^ = 0.391 *formula: numFam ∼* *groundDiv* *+* *groundCover* *+* *income* *+* *sqFeet* *+* *imp500m*				
(intercept)	3.39257	0.08091	41.93	<0.001
local ground vegetation diversity	0.22532	0.06238	3.612	<0.001
local ground vegetation coverage	0.30292	0.07075	4.282	<0.001
mean neighbourhood income	0.40011	0.08974	4.459	<0.001
house square footage	0.62026	0.07957	7.795	<0.001
impervious surface area at 500 m radius	0.16108	0.10105	1.594	0.110918
(*b*) BIC = 558.874; pseudo-*R*^2^ = 0.385 *formula: numFam* *∼* *groundDiv* *+* *groundCover* *+* *income* *+* *sqFeet*				
(intercept)	3.46595	0.06611	52.428	<0.001
local ground vegetation diversity	0.20631	0.06146	3.357	<0.001
local ground vegetation coverage	0.31647	0.07036	4.498	<0.001
mean neighbourhood income	0.3798	0.08897	4.269	<0.001
house square footage	0.57488	0.07456	7.71	<0.001
(*c*) AIC = 537.78; pseudo-*R*^2^ = 0.418 *formula: numFam ∼* *groundDiv* *+* *groundCover* *+* *income* *+* *sqFeet* *+* *groundCover × income*				
(intercept)	3.39257	0.08091	41.93	<0.001
local ground vegetation diversity	0.22418	0.06162	3.638	<0.001
local ground vegetation coverage	0.74517	0.13474	5.531	<0.001
mean neighbourhood income	0.98593	0.181514	5.325	<0.001
house square footage	0.59093	0.07487	7.892	<0.001
interaction (ground cover × income)	−1.12461	0.30257	−3.717	<0.001

Our finding, that indoor arthropod diversity increases in neighbourhoods with higher mean income, mirrors the ‘luxury effect’ found in studies of biodiversity in the urban outdoor environment [5–7,19]. In light of previous studies of the luxury effect [1–4] and our characterization of indoor arthropods [12], we hypothesize that affluence contributes to indoor arthropod richness by directly influencing plant coverage and diversity outdoors (at the neighbourhood level—as shown by references [1–4]), which in turn influences the prevalence of plant associated arthropods that then find their way indoors. In this scenario, our results suggest a broad ranging luxury effect that appears to cascade from choices made in landscaping and urban planning at the scale of city blocks to the indoor environments of individual houses.

Indoor biodiversity tends to be a mix of both human-associated (synanthropic) species and outdoor species that are inadvertently filtered from the surrounding landscape [12]. In respect of arthropods, houses act as exceptional traps: passively collecting like Malaise traps, but also acting as light and bait traps. Broadly speaking, the majority of indoor arthropods are flies, spiders, beetles and ants—groups that are also common in outdoor environments. These groups are often highly mobile and their survival often depends on outdoor vegetation [12]. These outdoor species occur in houses alongside species that directly depend on humans and/or the built environment (e.g. dust mites, pantry pests). Indoor arthropod diversity is, in part, a reflection of the world outdoors.

As expected, we found that outdoor vegetative ground cover and diversity in gardens of individual houses predicted indoor arthropod diversity; however, we did not find that houses in higher-income neighbourhoods necessarily had more vegetation in their individual gardens. To better understand the impact of vegetation on indoor arthropod diversity, we further explored the interactions between income and our house-level vegetation variables. The addition of an interaction term between neighbourhood income and house-level ground vegetation cover decreased AIC scores, indicating that this interaction term further improved our models (table 1*c*).

**Figure 2.**
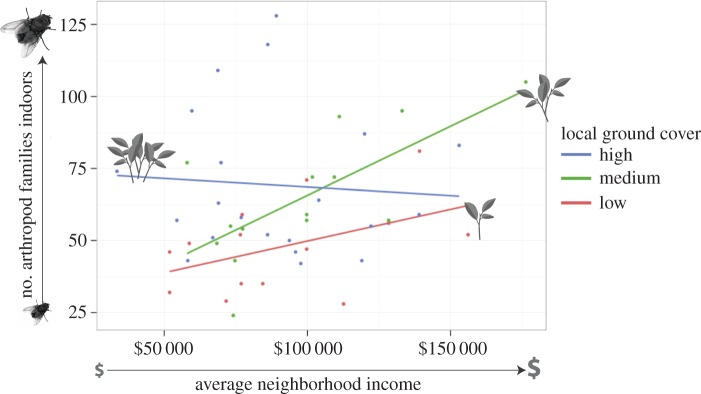
Interaction plot*.* For houses with low and medium levels of vegetative ground cover, neighbourhood income had a strong influence on number of arthropod families.

The interaction term revealed that for houses whose gardens have limited ground vegetation cover, being located in a higher-income neighbourhood had a strong positive effect on indoor arthropod diversity (figure 2). Yet for houses that have gardens with high ground vegetation cover, neighbourhood income did not influence indoor arthropod diversity. We suspect that in higher-income neighbourhoods, enhancements at the neighbourhood scale (including higher vegetation overall—as found in references [1–4]) can compensate for limited vegetation in the garden of an individual house. Thus, simply being located in a higher-income neighbourhood may provide ecological benefits to outdoor and indoor biodiversity. This suggests that vegetation at the scale of neighbourhoods can be predictive of indoor arthropod diversity at the scale of individual houses. It matters, in short, not only how much vegetation you have in your garden, but how much is present in the gardens and other habitats nearby [20].

Although arthropod diversity across houses differed in association with ecological and socioeconomic variables, we found, based on our community composition analyses**,** that the types of arthropods found indoors did not appear to vary substantially with these same variables (electronic supplementary material, table S1). For a breakdown of arthropod taxonomic diversity inside low, medium and high income houses, see electronic supplementary material, table S2.

A point of consideration regarding our results is that both our diversity and community composition analyses were unable to address potential confounding factors associated with house size—such as sampling effort and an increased number of microhabitats. Another caveat is that all of our participants were solicited voluntarily and only free-standing houses were included; thus, our sample is skewed toward middle and higher-income neighbourhoods (range: $33 510–176 288; mean: $92 337 ± 30 385). Further work covering a broader range of housing types, and neighbourhood demographics and vegetation metrics may expose other taxonomic and diversity patterns that are currently undetected.

As more of the planet becomes urbanized, the proportion of the ecological world potentially influenced by human socioeconomics will increase. Mixed responses to urbanization have been found for plants and animals—in part because of the confounding luxury effect [19]. The luxury effect has not previously been documented in respect of indoor environments (or even arthropods in outdoor environments for that matter), but it seems to be a response that cascades from affluence: increased vegetation at the neighbourhood scale leads to greater outdoor arthropod diversity, which translates to higher indoor arthropod diversity. Our unexpected, and perhaps counterintuitive finding of higher indoor arthropod diversity in wealthier neighbourhoods highlights how much we have yet to learn about indoor ecology.

## Supplementary Material

Supplementary Table 1

## Supplementary Material

Supplementary Table 2

## Supplementary Material

Supplementary Figure 1

## Supplementary Material

Main Dataset

## Supplementary Material

Community Composition Dataset

## Data accessibility

All data used for this study have been included as electronic supplementary material associated with this publication.
