# Supplementary figures and images for: Exoskeletons and economics: indoor arthropod diversity increases in affluent neighbourhoods
Source: Biol Lett. 2016 Aug;12(8):20160322. doi: 10.1098/rsbl.2016.0322 (PMC5014024; doi:10.1098/rsbl.2016.0322)

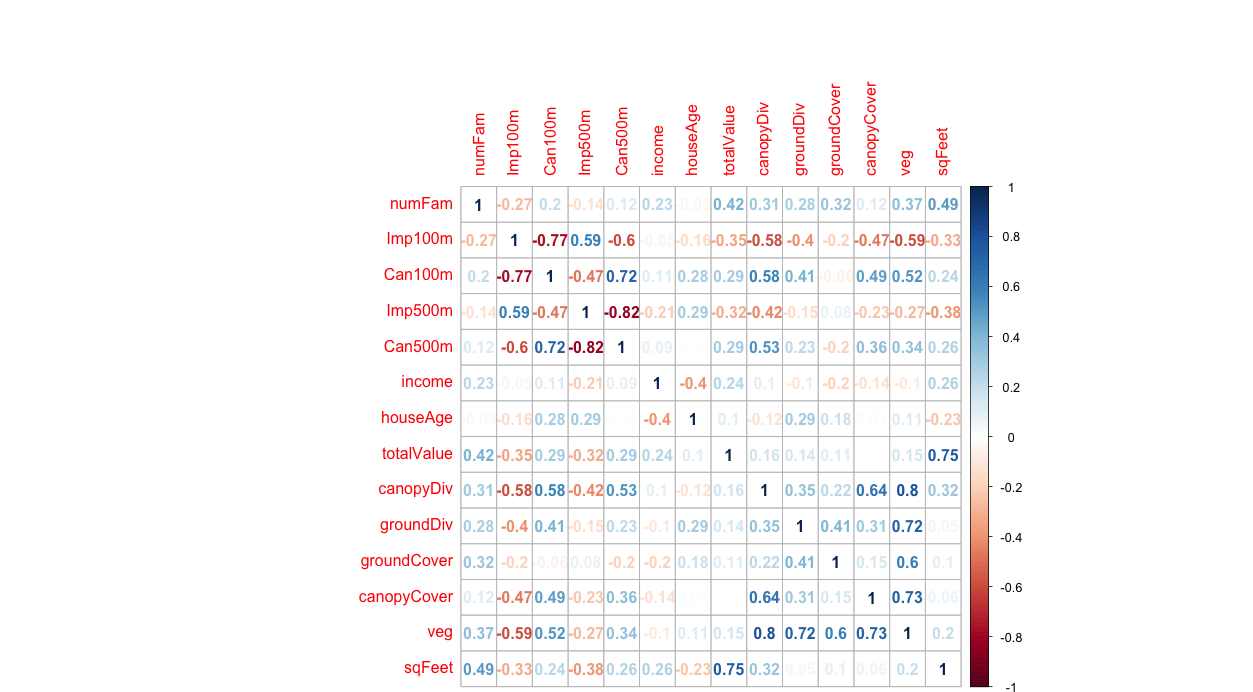

Supplement: Supplementary Figure 1 [file rsbl20160322supp3.tiff]
